# Buckling Analysis of Vacancy-Defected Graphene Sheets by the Stochastic Finite Element Method

**DOI:** 10.3390/ma11091545

**Published:** 2018-08-27

**Authors:** Liu Chu, Jiajia Shi, Shujun Ben

**Affiliations:** 1 School of Transportation, Nantong University, Nantong 226019, China; chuliu@ntu.edu.cn; 2 State Grid Nantong Power Supply Company, Nantong 226019, China; ntbenshj@jsepc.com

**Keywords:** buckling analysis, graphene sheets, vacancy defects, Monte Carlo-based finite element method

## Abstract

Vacancy defects are unavoidable in graphene sheets, and the random distribution of vacancy defects has a significant influence on the mechanical properties of graphene. This leads to a crucial issue in the research on nanomaterials. Previous methods, including the molecular dynamics theory and the continuous medium mechanics, have limitations in solving this problem. In this study, the Monte Carlo-based finite element method, one of the stochastic finite element methods, is proposed and simulated to analyze the buckling behavior of vacancy-defected graphene. The critical buckling stress of vacancy-defected graphene sheets deviated within a certain range. The histogram and regression graphs of the probability density distribution are also presented. Strengthening effects on the mechanical properties by vacancy defects were detected. For high-order buckling modes, the regularity and geometrical symmetry in the displacement of graphene were damaged because of a large amount of randomly dispersed vacancy defects.

## 1. Introduction

Graphene sheets is a promising nanomaterial with extraordinary properties for a wide range of applications. A great amount of academic research and experiments have been conducted to explore the properties of single-layer graphene sheets [1,2,3,4]. However, the existence of vacancy defects is unavoidable and difficult to predict. The large deviation observed in simulations and experiments has been attributed to the presence of defects in the nanotube structure [5,6]. Vacancy defects crucially impact on the mechanical properties of graphene sheets. Therefore, research on the vacancy defects of graphene sheets is essential to better understand the reasons of the described experimental deviations.

The main difficulty in the analysis of graphene’s mechanical behavior is the small size of this material [7,8]. It is hard to make accurate measurements in physical experiments at the nanometer scale, while analytical and numerical methods are powerful alternatives in research. The solid atomic-based methods are promising techniques in mechanical and electronic simulations, especially the molecular dynamics simulation [9] and the tight-binding molecular dynamics [10]. Besides, the density functional theory [11] is another powerful support. On the other hand, size-dependent continuum theories are also applied in the prediction and evaluation of graphene sheets, such as the non-local elastic theory [12], the modified couple stress theory [13], and the strain gradient theory [14]. However, when the number of atoms is large in a system, the atomic-based methods entail large computational expenses, whereas the continuum theories ignore or require complicated analysis to evaluate the defects or aleatory uncertainties presented in nanostructures. Therefore, it is necessary to develop an appropriate stochastic finite element method to introduce the randomly distributed vacancy defects in the deterministic finite element method.

In this challenging field, effective attempts and efforts have been made to analyze the influence of different defects. Banhart et al. [15] pointed out that a small amount of defects in the atomic structure of nanomaterials can profoundly affect the mechanical and electronic properties of graphene sheets. To this end, molecular dynamics [16] was used to analyze the influence of vacancy defects on the properties of a material. By the same method, the Stone–Wales defects of carbon nanotubes under tension were evaluated [17,18]. However, the appropriate quantification of the influence of vacancy defects of graphene sheets on dynamic non-linear processes, such as free vibration and buckling, is still facing problematic [19]. The stochastic and unpredictable properties of vacancy defects and their sophisticated effects on the mechanical responses of graphene sheets are unsolved problems, which deserve more attention.

The Monte Carlo simulation (MCS), a sophisticated sampling method, can be used in program design and has been widely adopted in various fields of research including engineering [20,21,22,23]. When the sampling space is large enough, the MCS can achieve an acceptable level of accuracy in numerical results. Normally, the results of MCS are set as comparison criteria or precise results [24,25]. In addition, the combination of the MCS and the finite element analysis is feasible and convenient. This paper combines the MCS with the finite element method (FEM) to form a stochastic finite element method called Monte Carlo-based finite element method (MC–FEM), by which the random dispersed vacancy defects can be successfully propagated and simulated in graphene sheets.

In this paper, the elastic buckling characteristics of single-layer graphene sheets are studied, and the effects of random vacancy defects are considered. In Section 2, the honeycomb lattice of graphene sheets is introduced and explained, and the buckling analysis of vacancy-defected graphene sheets is realized by the MC–FEM; an example of graphene sheets with random dispersion vacancy defects is also presented. Section 3 provides a discussion about the probability results based on mathematical statistics; the comparison of different amounts of vacancy defects is also presented in this section, and the vector sum of the displacement of graphene sheets with the vacancy defects is depicted in contour figures. In the last section, a brief conclusion is given.

## 2. Materials and Methods

### 2.1. Elastic Buckling

**Figure 1:**
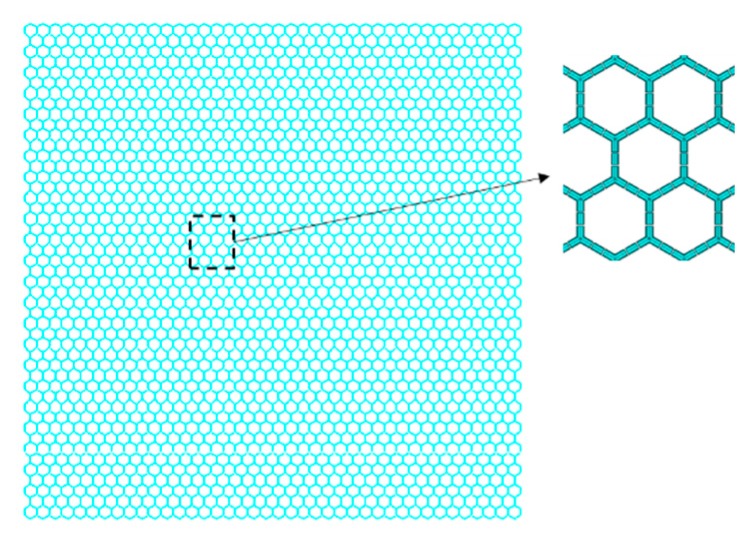
Graphene sheets with beam finite elements.

Since the carbon atoms in graphene sheets are combined with covalent bonds to form a honeycomb 2D lattice, the displacement of a single atom is the response of graphene sheet to external forces. The deformation and displacement of the carbon atoms are constrained by the C–C bonds. Therefore, modelling the C–C bonds in graphene sheets is an appropriate method to analyze the mechanical reaction and response in both static and dynamic states. Considering its technological development and sound mathematical foundation, the efficacy of FEM in the nanomaterial computation field was explored. The beam element was chosen as the finite element in the elastic buckling analysis of graphene sheets, as presented in Figure 1.

For the elastic buckling analysis, the eigenvalue equation for the structure subjected to static load is expressed as

(1) $$\left([K]\;+\lambda_{i}\left[S\right]\right){\left\{\psi \right\}}_{i}=\left\{0\right\}$$

where [K] and [S] are the stiffness matrices and stress stiffness matrices, respectively; $\lambda_{i}$ and $\psi_{i}$ are the i-th eigenvalue and eigenvector of displacement. With the external compressive or tensile force, the eigenvector displacement cannot be zero. Therefore, the solution of the above equation can be written as

(2) $$\left|[K]\;+\lambda_{i}\left[S\right]\right|=0$$

In the finite element model of graphene lattice, the global stiffness matrix [K] is obtained from the element stiffness matrix $K^{e}$

(3) $$K^{e}=\left[\begin{array}{cc} k_{ii\;} & k_{ij}\\ k_{ji} & k_{jj} \end{array}\right]$$

(4) $$k_{ii\;}=\left[\begin{array}{cccccc} AE/a & 0 & 0 & 0 & 0 & 0\\ 0 & 12EI_{y}/a^{3} & 0 & 0 & 0 & 6EI_{z}/a^{2}\\ 0 & 0 & 12EI_{y}/a^{3} & 0 & -6EI_{y}/a^{2} & 0\\ 0 & 0 & 0 & GJ/a & 0 & 0\\ 0 & 0 & -6EI_{y}/a^{2} & 0 & 4EI_{y}/a & 0\\ 0 & 6EI_{z}/a^{2} & 0 & 0 & 0 & 4EI_{z}/a \end{array}\right]$$

(5) $$k_{jj\;}=\left[\begin{array}{cccccc} AE/a & 0 & 0 & 0 & 0 & 0\\ 0 & 12EI_{y}/a^{3} & 0 & 0 & 0 & -6EI_{y}/a^{2}\\ 0 & 0 & 12EI_{y}/a^{3} & 0 & 6EI_{y}/a^{2} & 0\\ 0 & 0 & 0 & GJ/a & 0 & 0\\ 0 & 0 & 6EI_{y}/a^{2} & 0 & 4EI_{y}/a & 0\\ 0 & -6EI_{z}/a^{2} & 0 & 0 & 0 & 4EI_{y}/a \end{array}\right]$$

(6) $$k_{ij\;}=\left[\begin{array}{cccccc} -AE/a & 0 & 0 & 0 & 0 & 0\\ 0 & -12EI_{y}/a^{3} & 0 & 0 & 0 & 6EI_{z}/a^{2}\\ 0 & 0 & -12EI_{y}/a^{3} & 0 & -6EI_{y}/a^{2} & 0\\ 0 & 0 & 0 & -GJ/a & 0 & 0\\ 0 & 0 & 6EI_{y}/a^{2} & 0 & 2EI_{y}/a & 0\\ 0 & -6EI_{y}/a^{2} & 0 & 0 & 0 & 4EI_{y}/a \end{array}\right]$$

where AE is the axial stiffness, $EI_{z}$ is the in-plane bending stiffness, $EI_{y}$ is the out-of-plane bending stiffness, GJ is the torsional stiffness, and a is the length of the beam element.

Then, the critical buckling stress $\sigma_{cr}$ can be computed by

(7) $$\sigma_{cr\;}=\lambda_{cr}\sigma_{r}$$

where $\lambda_{cr}$ is the first eigenvalue, and $\sigma_{r}$ is the original external compressive stress.

### 2.2. Graphene Sheets

The validation of the modified Morse potential has been proved in previous studies to predict the mechanical properties of carbon nanotubes. The potential was successfully employed in the nonlinear response simulation of nanomaterials under tensile and torsional external forces [26]. The effects of defects on the Young’s modulus of nanotubes were investigated using the modified Morse potential [27]. In comparative studies, the modified Morse potential provides more precise prediction results than the reactive empirical bond-order potential [28,29]. As stated in a previous work, the potential energy of the entire graphene sheets is written as [30]

(8) $$\begin{array}{l} E=E_{s}+E_{a}\\ E_{s}=D\left[{\left(1-e^{-\beta \left(r-r_{{\;}_{0}}\right)}\right)}^{2}-1\right]\\ E_{a}=\frac{1}{2}k_{\theta }{\left(\theta -\theta_{0}\right)}^{2}\left[1+k_{s}{\left(\theta -\theta_{0}\right)}^{4}\right] \end{array}$$

where $E_{s}$ is the bond energy due to the bond stretching, $E_{a}$ is the bond energy corresponding to the angle bending, r is the bond length, and θ is the angle of the adjacent bond. The parameters of the potential are [30]

(9) $$\begin{array}{l} r_{0}=1.42\times 10^{-10\;}m,D=6.031\times 10^{-19}\mathrm{Nm},\beta =2.625\times 10^{-10}m^{-1}\\ \theta_{0}=2.094\mathrm{rad},k_{\theta }=0.9\times 10^{-18}{\mathrm{Nm}/\mathrm{rad}}^{2},k_{s}=0.754{\mathrm{rad}}^{-4} \end{array}$$

On the basis of the above expression, the diameter d, Young’s modulus E, and shear modulus G of the beam elements in the honeycomb lattice of graphene sheets can be calculated according to

(10) $$\{\begin{array}{c} d=4\sqrt{\frac{k_{\theta }\;}{k_{r}}}\\ E=\frac{k_{r}^{2}L}{4\pi k_{\theta }}\\ G=\frac{k_{r}^{2}k_{\tau }L}{8\pi k_{\theta }^{2}} \end{array}$$

where $k_{r},k_{\theta },k_{\tau }$ are the bond-sketching, bond-bending, and torsional resistance force constants, respectively. 

### 2.3. Graphene Sheets with Vacancy Defects

As mentioned above, each beam in the FEM of graphene sheets has a specific number. If a number is chosen in the MCS, a corresponding beam is removed from the entire hexagon structure of graphene sheets. The defect density, *Per*, can be expressed as

(11) $$Per=\frac{D_{n}\;}{A_{n}}$$

where $D_{n}$ is the number of vacancy defects, and $A_{n}$ is the total number of beams in the FEM of pristine graphene sheets.

**Figure 2:**
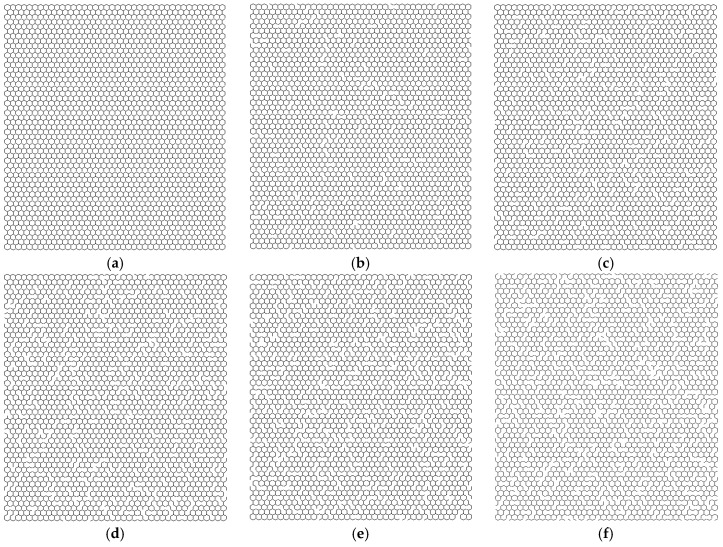
Specific examples of the graphene sheets with vacancy defects. The samples (**a**–**f**) have 0%, 2%, 4%, 6%, 8%, and 10% of vacancy defects, respectively.

For the parameters related to the material and geometrical properties, the Young’s modulus and Poisson’s ratio in graphene sheets are settled as 1.2 TPa and 0.2, respectively; the length of the bond in the honeycomb lattice is 0.27 nm, and the diameter in the cross section of the beam finite element is 0.032 nm, according to the literature [6]. For the pristine graphene sheets, there are 6226 beams and 16,664 nodes created in the deterministic FEM. For the boundary condition, the six degrees of freedom for key points in the two longitudinal edges are all supposed to be zero. Each key point in the two transverse edges receives unit force in tension. Figure 2 presents the specific examples of graphene sheets with different *Per*. By the MCS, the vacancy defects are randomly distributed in graphene sheets. 

When the number of samples is increased, the relative errors of MCS are obviously reduced. In other words, when the sampling space is large enough, the MCS can reach an acceptable accuracy. However, the required large sampling sets and repetitions of FEM significantly increase the costs of experimentation. Evidently, a trade-off exists between the result accuracy of MCS and the computational costs in numerical simulations. On the basis of a previous work [24], the number of the MCS repetitions is set to 500 in the stochastic finite element method.

After validation of the FEM, the MC–FEM was applied until sufficient sampling was completed. By the MCS generator, the beams representing vacancy defects were chosen and removed from the pristine graphene sheets. The elastic buckling analysis was performed by the MC–FEM in the vacancy-defected graphene sheets.

## 3. Results and Discussion

### 3.1. Probability Analysis

Given that vacancy defects are randomly distributed in graphene sheets, it is hard to evaluate or predict with certainty their locations throughout the graphene sheets. When the number of vacancy defects, *Per*, is determined, one application of the MC–FEM is insufficient to describe the stochastic placement of the vacancy defects. It was, therefore, necessary to repeat the MCS and implement the FEM for a sufficient number of times for as thorough an analysis as possible.

Table 1 demonstrates the statistical results of the elastic buckling after repeating the MC–FEM. The average results of the critical buckling stress were more reliable and could be used to study the buckling behavior of vacancy-defected graphene sheets. Obviously, the mean of critical buckling changed nonlinearly with the increase of the vacancy defects. When the *Per* exceeded 3%, the critical buckling stress decreased suddenly. When the amount of vacancy defects was minor, the reduction was slow and insignificant.

When *Per* was equal to 2%, the reduction rate of the critical buckling stress was 1.64% compared with graphene without defects. The influence of the vacancy defects on the elastic buckling behavior was not obvious and could be ignored. However, when *Per* was 3%, 4%, and 5%, the reduction of the critical buckling stress was 9.91%, 21.7%, and 44.07%, respectively, compared with graphene without defects. Therefore, when the vacancy defects exceeded a certain density, the vacancy defects had a great influence on the elastic buckling behavior of graphene. The existence of vacancy defects damages the structural symmetry and integrity of the graphene and has a deep effect on the buckling behavior.

As shown in Table 1, the critical buckling stress decreased by different degrees with the increase of vacancy defects. These results regard the randomly dispersed vacancy positions in graphene sheets on the basis of the statistical results. Furthermore, the standard variance of the critical buckling stress increased with the increase of vacancy defects. When the vacancy defect percentage exceeded 3%, the standard variance of the critical buckling stress also increased sharply.

**Figure 3:**
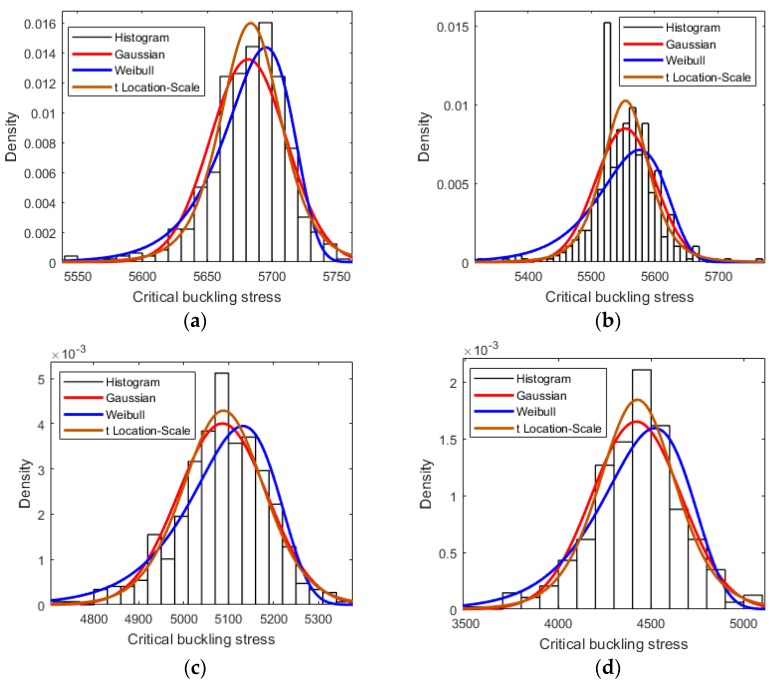
The probability density distribution for different amounts of vacancy defects. The samples (**a**–**d**) represent 1%, 2%, 3%, and 4% of vacancy defects, respectively.

Because of the randomly distributed placement of the vacancy defects in graphene sheets, the critical buckling stress was variable and fluctuated within certain ranges. Figure 3 shows the probability density distribution of the critical buckling stress for graphene sheets with 1%, 2%, 3%, and 4% of vacancy defects, respectively. It is clear that, for different amounts of vacancy defects, the histogram of the probability density distribution was close to the Gaussian or t Location-Scale distributions. The Gaussian or t Location-Scale distribution was more accurate for the description of the uncertainty and the effects caused by stochastic vacancy defects. 

**Figure 4:**
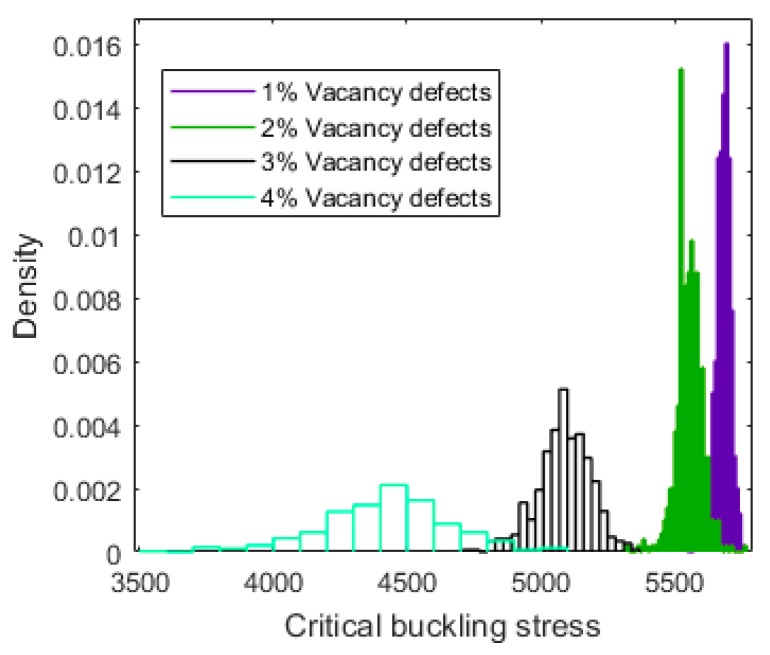
Comparison of the probability density for graphene sheets with different amounts of vacancy defects.

**Figure 5:**
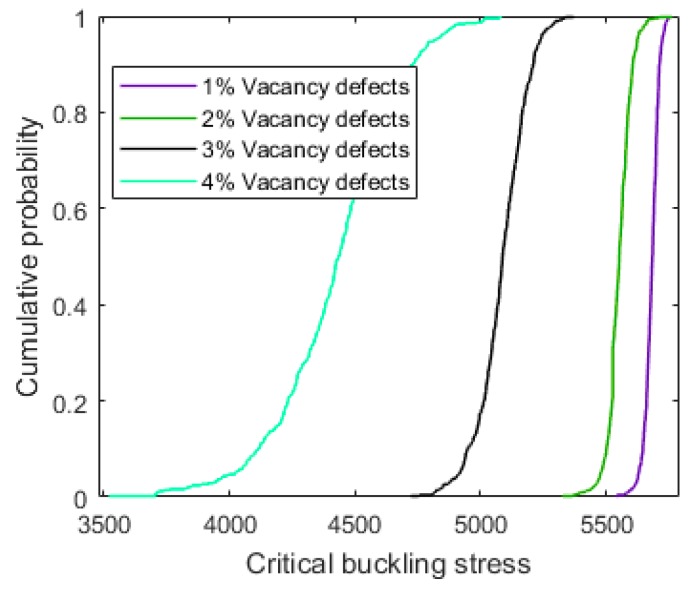
Comparison of the cumulative probability for graphene sheets with different amounts of vacancy defects.

In order to discuss the probability results more clearly, Figure 4 and Figure 5 present the comparison of the probability density distribution and cumulative probability for graphene sheets with different vacancy defect percentages. It is obvious that when the vacancy defect amount was small, the influence of the randomly dispersed location of the vacancy defects on the buckling behavior was weak. As shown in Figure 4, the probability density distribution of graphene sheets with 1% of vacancy defects was more concentrated than that of graphene sheets with 4% of vacancy defects. Figure 5 also confirms this point. In synthesis, with the increase of vacancy defects, the critical buckling stress of graphene sheets was distributed in a larger interval with greater variance, due to the influence of the stochastically dispersed vacancy defects. 

### 3.2. Comparison and Discussion

**Figure 6:**
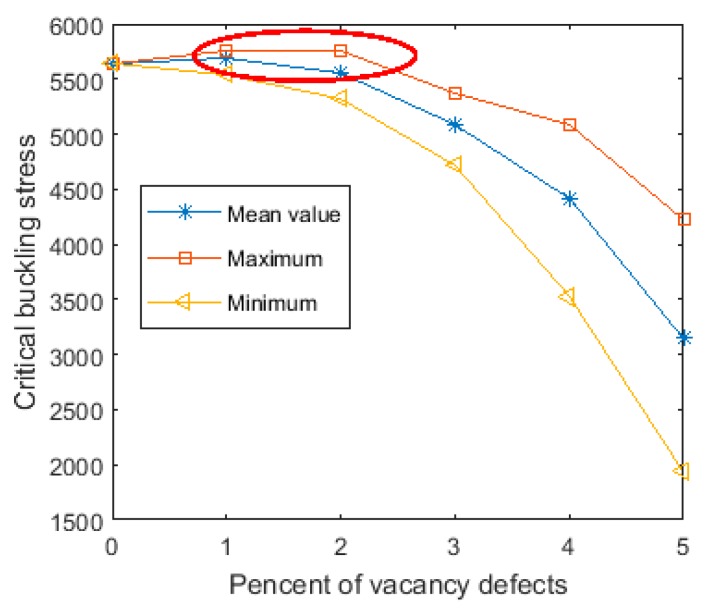
Interval results of MC–FEM.

The probability analysis of the buckling behavior of vacancy-defected graphene sheets was successfully performed based on the results of the MC–FEM. Besides the probability results, the range of values of the critical buckling stress was determined in this study. From these results, the extreme situations and the corresponding critical buckling stress were analyzed and are shown in Figure 6. When the amount of the vacancy defects was tiny, the maximum, the minimum, and the mean buckling stress were clustered together or concentrated in a narrow range. These observations confirm the results of the probability density distribution and the cumulative probability results depicted in Figure 4 and Figure 5.

In addition, the stiffness strengthening effect was observed in the curve of the maximum critical buckling stress when the amount of vacancy defects was smaller than 2%, as reported in Figure 6. With the appearance of vacancy defects, the reduction of the critical buckling stress varied. The critical buckling stress augmented when *Per* was small. The curve of the maximum critical buckling stress was not as smooth as those of the minimum and the mean critical buckling stress. It is clear that the curve of the maximum critical buckling stress was characterized by two different stages. In the first stage, the critical buckling stress became larger with the increase of vacancy defects. However, in the second stage, the critical buckling stress sharply reduced with the increase of vacancy defects. This phenomenon was also measured and tested in physical experiments [31], and the results are in good agreement with those obtained by the MC–FEM.

**Figure 7:**
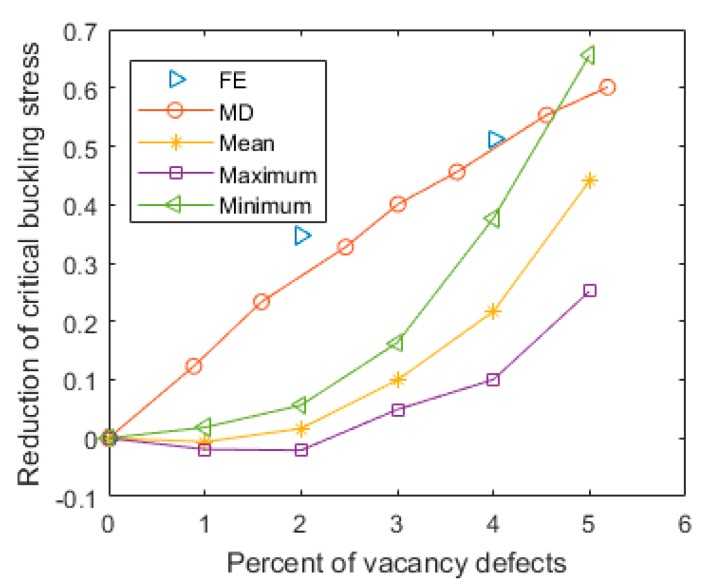
Comparison with results reported in the literature [32] (with 95% confidence interval). FE: finite element; MD: molecular dynamics.

Furthermore, with the augmentation of the number of vacancy defects, not only the critical buckling stress itself was affected, but also the interval between the maximum and minimum values of the MC–FEM was amplified. In Figure 7, the results of the presented method are compared with those of the molecular dynamics (MD) and finite element (FE) methods in reported the literature [32]. This figure shows that the values measured in this study are generally smaller than those obtained by the MD and discrete FE methods, especially when the vacancy defect amount was small. The explanation for this deviation is based on how the vacancy defects are identified. In this study, the number of vacancy defects corresponds to the number of vacancy beams in the hexagon lattice of graphene sheets, while in the study using the MD method, the absence of atoms is recorded as vacancy defects. One atom of vacancy leads to the loss of three neighbor bonds. Besides, in this study, the vacancy defects are dispersed in the entire graphene randomly, whereas in the previous study [32], the vacancy defects were periodically and regularly distributed. Therefore, it is reasonable to affirm that the periodic atom vacancy defects have more distinct effects on the critical buckling stress of graphene sheets than the stochastic beam vacancy defects.

Similar to this study, kinetic lattice Monte Carlo simulation [33] was used to study the evolution of vacancy defects in the entire graphene. By implementing ab initio energetics, the quantitative computation of the system kinetics, the morphology of defects, and their interactions were analyzed. The small aggregates and vacancy defects had an evident influence on the nucleation stage of graphene. The vacancy defects in graphene were hampered by the relatively large barrier generated by the vacancy-surrounding strain field [34]. Therefore, the elastic buckling analysis of graphene is not only important to the mechanical properties of the material, but also applicable to the kinetic vacancy evolution process.

The buckling of graphene is feasible and appropriate in the application of hydrogen storage and memcapacitor. The possibility of recruiting the buckled membrane as a plate of capacitor with memory was validated by MD simulations and elastic mechanical calculations [35]. Besides, the storage and release of hydrogen were implemented in buckling graphene with convex and concave regions [36]. The buckling graphene is a revisable and environmentally friendly method of energy storage. Hydrogen chemisorption is energetically favored in convex regions of graphene, whereas concave regions of graphene are more propitious to hydrogen release. By controlling the deformation of the elastic buckling in graphene, the dynamical process can be successfully conducted. The vacancy defects in graphene sheets can amplify the displacement and deformation of the graphene sheets in specific locations. The study of the elastic buckling behavior of graphene with vacancy defects is promising to improve the efficiency of hydrogen storage.

### 3.3. Displacement Results of Graphene Sheets

**Figure 8:**
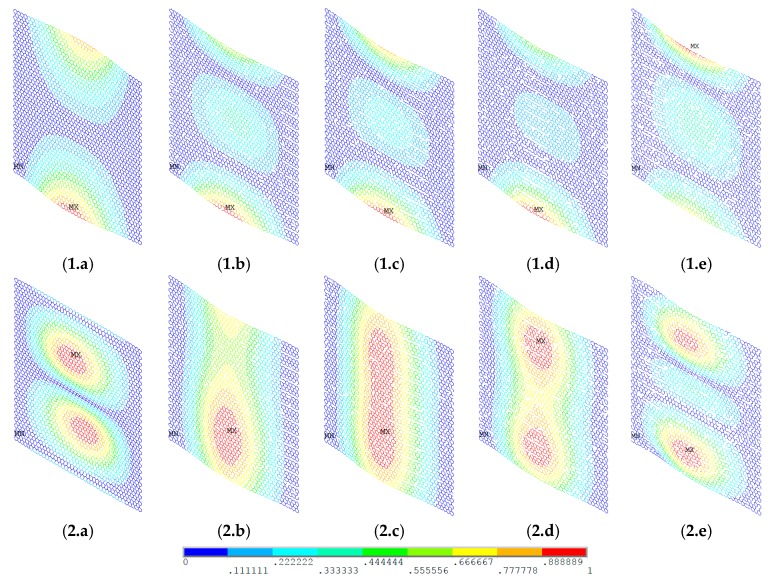
Vector sum of the displacement of graphene sheets. (**1.a**–**1.e**) First-order buckling mode; (**2.a**–**2.e**) fourth-order buckling mode; (**a**–**e**) 0%, 1%, 2%, 3%, and 4% of vacancy defects, respectively.

**Figure 9:**
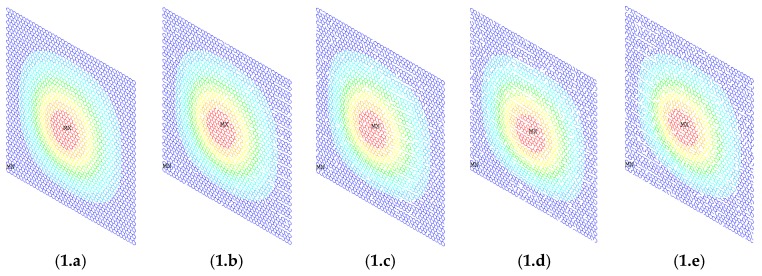

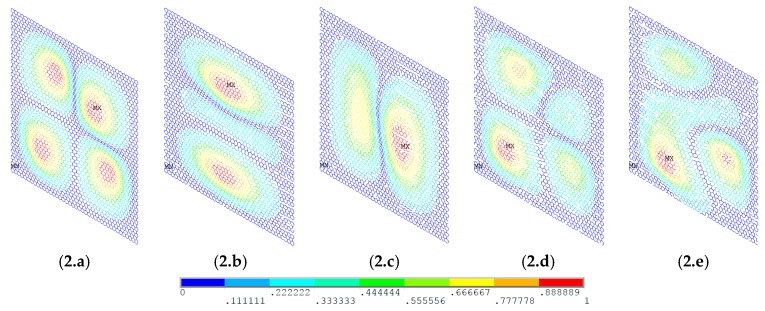
Vector sum of the displacement of graphene sheets. (**1.a**–**1.e**) First-order buckling mode; (**2.a**–**2.e**) fourth-order buckling mode; (**a**–**e**) 0%, 1%, 2%, 3%, and 4% of vacancy defects, respectively.

In order to demonstrate the buckling behavior of vacancy-defected graphene sheets, Figure 8 and Figure 9 provide the vector sum results of the displacement in the first- and the fourth-order buckling modes. The boundary condition in Figure 8 is as mentioned above. The six degrees of freedom for key points in the two longitudinal edges were all supposed to be zero. For each key point in the two transverse edges, there was unit force in tension. In Figure 9, the situation of two longitudinal edges is the same as in Figure 8. Not only the key points in the two transverse edges had unit force in tension, but also the rotations in each key points were limited.

With the increase of the number of vacancy defects, the vector sum of the displacement of the low-order buckling mode maintained geometrical symmetry. A deviation was not very evident, which as shown in Figure 8(1.a–1.e) and Figure 9(1.a–1.e). However, for the high-order buckling mode, the results were totally different. Depending on the randomly distributed location of the vacancy defects in graphene sheets, Figure 8(2.b–2.e) are quite different from Figure 8(2.a). In Figure 9, the uncertainty in vacancy defects eliminated the regular and geometrical symmetry.

For the deformation of the elastic buckling, the results of this study are in good agreement with quasi-static MD predictions. The buckling surfaces of highly cross-linked epoxy polymers under stress obeyed the paraboloid yield criterion at different temperatures [37]. Besides, by quasi-static simulations, the yield strength of the amorphous glassy polyethylene was computed in a hierarchical multiscale model with temperature and strain rate dependence [38]. Transmission electron microscopy revealed that the buckling wavelengths were 3.6 ± 0.5 and 6.4 ± 0.8 Å in graphene, respectively [39]. There were only several (two or three) unit cells in the major buckling direction. Furthermore, the orientation of the lowest deformation energy was spontaneously chosen in the buckling process. The Euler buckling theory is appropriate in computing the deformation and displacement of graphene. Therefore, the MC–FEM is effective in analyzing the influence of randomly dispersed vacancy defects in the elastic buckling of graphene.

## 4. Conclusions

In this paper, a detailed study of the elastic buckling of rectangular graphene sheets with different amounts of vacancy defects was carried out using the MC–FEM. The random dispersion of vacancy defects in graphene was taken into consideration, and the effects of the amount of vacancy defects and stochastically distributed placements were discussed. From the proposed MC–FEM, the following conclusion can be drawn:

With the increase of vacancy defects in graphene, the critical buckling stress sharply decreased, and the standard variance for the buckling stress was evidently amplified when the number of vacancy defects exceeded 3%. When the vacancy defect percentage was equal to 5%, the reduction of the critical buckling was as large as 44%. The vacancy defects profoundly influenced the buckling behavior of the graphene lattice. With the increase of vacancy defects, the geometrical symmetry in the vector sum of displacement was obviously affected.

The randomly distributed placement of vacancies caused a fluctuation and deviation in the buckling behavior of the graphene vibration. The intervals between the maximum and minimum values were amplified with the increase of vacancy defects. For different amounts of vacancy defects, the probability density distributions of the critical buckling stress were close to the Gaussian or t Location-Scale distributions.

Furthermore, the stiffness strengthening effect of the vacancy defects on graphene was discussed in this study. The possibility of improving the mechanical properties of graphene by vacancy defects was confirmed.

## Figures and Tables

**Table 1:** Statistical results of the elastic buckling for the Monte Carlo-based finite element method MC–FEM.

*Per* (%)	Mean (THz)	Variance^0.5	Skewness	Kurtosis
1	5.6817	0.0294	−0.0009	0.0053
2	5.5534	0.0471	−0.0003	0.0056
3	5.0863	0.0996	−0.0004	0.0035
4	4.4207	0.2416	−0.0003	0.0037
5	3.1576	0.4646	−0.0003	0.0029
